# Gender differences in obstructive sleep apnea syndrome: a pilot study

**DOI:** 10.1007/s11325-024-03052-x

**Published:** 2024-05-08

**Authors:** Antonio Fabozzi, Federico Pasqualotto, Marianna Laguardia, Pietro Francesco Natuzzi, Rosaria Capone, Alessia Steffanina, Daniela Pellegrino, Federica Olmati, Caterina Antonaglia, Paolo Palange

**Affiliations:** 1 Department of Public Health and Infectious Diseases, Pulmonology Unit, Policlinico Umberto I, “Sapienza” University of Rome, Viale del Policlinico, 155 Rome, Italy; 2 Pulmonology Department, University Hospital of Cattinara, Trieste, Italy

**Keywords:** Obstructive sleep apnea, Sleep apnea syndromes, Polysomnography, Continuous positive airway pressure, Disorders of excessive somnolence

## Abstract

**Purpose:**

OSAS is a syndrome that often presents clinically differently between men and women. The aim of this study was to assess the clinical presentation, nocturnal home sleep cardiorespiratory monitoring and therapeutic adherence to CPAP in both sexes to identify the most frequent patterns.

**Methods:**

Data from the first visit, the nocturnal home sleep cardiorespiratory monitoring and follow-up visit of 74 OSA patients were collected. Exclusion criteria included other respiratory and/or neuromuscular diseases (including Obesity hypoventilation syndrome) and other non-respiratory sleep disorders.

**Results:**

Men were older and had a higher supine AHI and ODI compared to women. In addition, BMI and age correlated positively with AHI in males. Women had a higher hypopneas frequency and better therapeutic adherence to CPAP.

**Conclusions:**

Men were associated with a higher AHI when sleeping in the supine position and this may be useful to look for new therapeutic options in combination with or as an alternative to CPAP. BMI correlated positively with AHI in men and this should be considered to stimulate weight loss as the main treatment to reduce the number of apneas/hypopneas, as men also had less therapeutic adherence to CPAP in our study. Females presented a significantly higher frequency of hypopneas than men, as well as a lower number of desaturation events per hour (ODI): these differences in the nocturnal home sleep cardiorespiratory monitoring could reflect different pathophysiological mechanisms of OSAS onset between the two sexes, which should be investigated in future scientific studies.

## Introduction

Obstructive sleep apnea syndrome (OSAS) is a condition characterised by repeated episodes of upper airway collapse during sleep. OSAS has always been considered a male-predominant syndrome, which is why scientific studies in the past mainly involved men. To this date, it is true that epidemiological data suggest a higher prevalence in males [1], but in the elderly the prevalence becomes similar between the two sexes [2]. Consequently, in recent decades, more and more scientific studies have focused on investigating the syndrome in females as well. But the epidemiological and pathophysiological data on the gender difference are still controversial today. For example, part of the literature reports that women with OSAS are more frequently obese than men, especially in cases of moderate obesity (BMI ≥ 35 kg/m^2^) [3]. However, there are studies from the literature claiming that BMI could not be used as a discriminating anthropometric factor for the gender difference in OSAS, as it does not differ significantly between the two sexes [4]. The AHI (apnoea-hypopnoea index), a parameter commonly used for diagnosing and stratifying the severity of OSAS, is also controversial in the literature when it is differentiated between the two genders. In fact, women generally have a lower AHI compared to men [5]. But there are as many studies in literature stating that the AHI is not sufficient to differentiate severity between the genders, as it is often similar in the two populations [6]. Pathophysiologically, hypotheses have been advanced to explain the different predisposition of men and women to the development of sleep apnoea. In fact, although there is still controversy today, it has been shown that men have a greater tendency to collapsibility of oropharyngeal musculature during sleep because the airways are longer and the soft tissues of the lateral pharyngeal wall are larger in men [7]. The men's pharynx is larger than the female's but tends to be more collapsible; indeed, female's pharynx is stiffer and less susceptible to collapse despite a smaller size [8]. Recent data on gender differences in collapsibility and upper airway resistance during sleep in patients with OSAS are lacking in literature.

The aim of our study is to investigate, in a real-life population followed at an expert centre for sleep breathing disorders, the differences between the two genders about parameters that are still discordant in literature such as age, BMI, polygraphic indicators, excessive daytime sleepiness, and therapeutic adherence to CPAP. The aim was therefore to verify what may be the discriminating factors in the gender difference in OSAS and consequently which factors should be focused on most in men and women, both for preventive and therapeutic purposes.

## Methods

This is a retrospective single-centre real-life observational study performed at the respiratory sleep disorder centre of Pneumology, Policlinico Umberto I, Rome, Italy. Data from the first visit, nocturnal HSCM (home sleep cardiorespiratory monitoring) and follow-up visit of 74 patients performed from January 2020 to January 2024 were collected. HSCM was conducted according to the standards of the American Academy of Sleep Medicine (AASM) [9] using SOMNO touch RESP device and DOMINO light software [10]. HSCM consisted of eight integrated channels: nasal flow, snore, thoracic effort, abdomen effort, oxygen saturation SpO2, pulse rate, plethysmogram and body activity. Inclusion criteria included age ≥ 18 years and a diagnosis of OSAS (AHI > 5 events per hour with disability-related symptomatology or AHI > 15 events per hour). Exclusion criteria included the absence of other respiratory and/or neuromuscular diseases, a central AHI ≥ 5 events per hour, a diagnosis of other non-respiratory sleep disorders, a diagnosis of Obesity hypoventilation syndrome (OHS). The following parameters were assessed: age, BMI, Mallampati score, Epworth sleepiness scale (ESS), AHI, prevalence of positional obstructive sleep apnoea (pOSA), supine AHI, nadir SpO2% (lowest value), hypopneas frequency compared to total events, Oxygen-desaturation index (ODI), mean desaturation duration, CPAP using rate and mean therapeutic pressure applied. Apnea was scored when there was a drop in the peak signal excursion by ≥ 90% of pre-event baseline using a nasal flowmeter sensor for ≥ 10 s [9]. Hypopnea was scored when the peak signal excursions drop by ≥ 30% of pre-event baseline using a nasal flowmeter sensor for ≥ 10 s in association with ≥ 3% arterial oxygen desaturation [9].

The diagnosis of pOSA was made with a supine AHI/non-supine AHI ratio ≥ 4 [11].

Statistical analysis: results are presented as mean ± standard deviation (SD). One-way analysis of variance (ANOVA) was used to compare the mean of variables and Pearson's correlation coefficient to measure the strength of linear association between variables. The chi-square test was used to compare the prevalence of variables in the two populations. The level of statistical significance was set at *p* < 0.05. Data analyses were conducted using Jamovi software (Table 1).

**Table 1:** Parameters and patients’ characteristics considered during the study

	Men (*n* = 40)	Women (*n* = 34)	*p* value
Age, years	68 ± 10	61 ± 10	F = 7.4; *p* = 0.008
BMI, kg/m^2^	30 ± 6	30 ± 6	NS
Mallampati	3 ± 1	3 ± 1	NS
ESS	7 ± 5	8 ± 6	NS
AHI, events per hour	26 ± 16	23 ± 15	NS
Supine AHI, events per hour	35 ± 20	19 ± 12	F = 16; *p* = 0.0001
pOSA prevalence, %	12%	14%	NS
Hypopneas frequency, %	43 ± 22%	58 ± 20%	F = 9; *p* = 0.003
Nadir SpO2, %	76.5 ± 13%	78 ± 10	NS
ODI, events per hour	27 ± 19	19 ± 12	F = 4.2; *p* = 0.04
Mean desaturation duration, sec	31 ± 6	30 ± 8	NS
CPAP using rate, %	47%	76%	χ = 5; *p* = 0.02
Mean therapeutic pressure, cmH2O	10.8 ± 2.6	9.8 ± 3.5	F = 0.9; *p* = 0.3

Values are means ± standard deviation

*BMI* body-mass index, *ESS* Epworth sleepiness scale, *AHI* apnoea-hypopnoea index, *pOSA* positional obstructive sleep apnoea, *ODI* oxygen desaturation index, *CPAP* continous positive airway pressure, *F* F ratio, *χ* chi-square, *NS* not significant

## Results

**Fig. 1:**
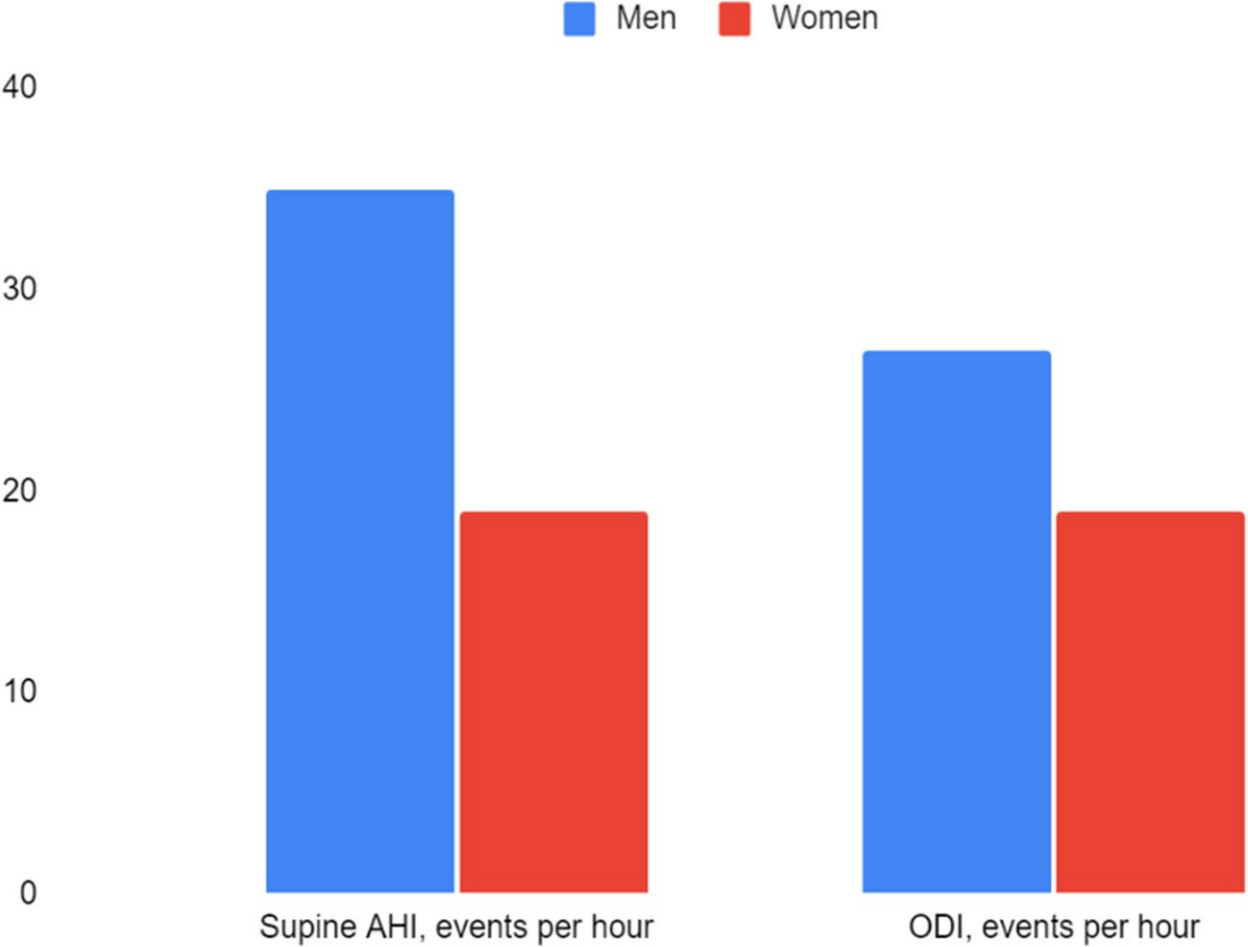
Significantly gender differences about supine AHI and ODI

74 patients (34 women and 40 men) were evaluated (Table 1). Men were older than women (*p* = 0.008), with a similar BMI (30 ± 6 kg/m2). The AHI did not differ significantly between the two groups while, stratifying AHI by position during sleep, the supine AHI was higher in men (Fig. 1, *p* = 0.0001). Although this difference of supine AHI, the rate of patients diagnosed with pOSA was similar between the two sexes (14% for men and 12% for women, χ = 0.07, *p* = 0.8).

**Fig. 2:**
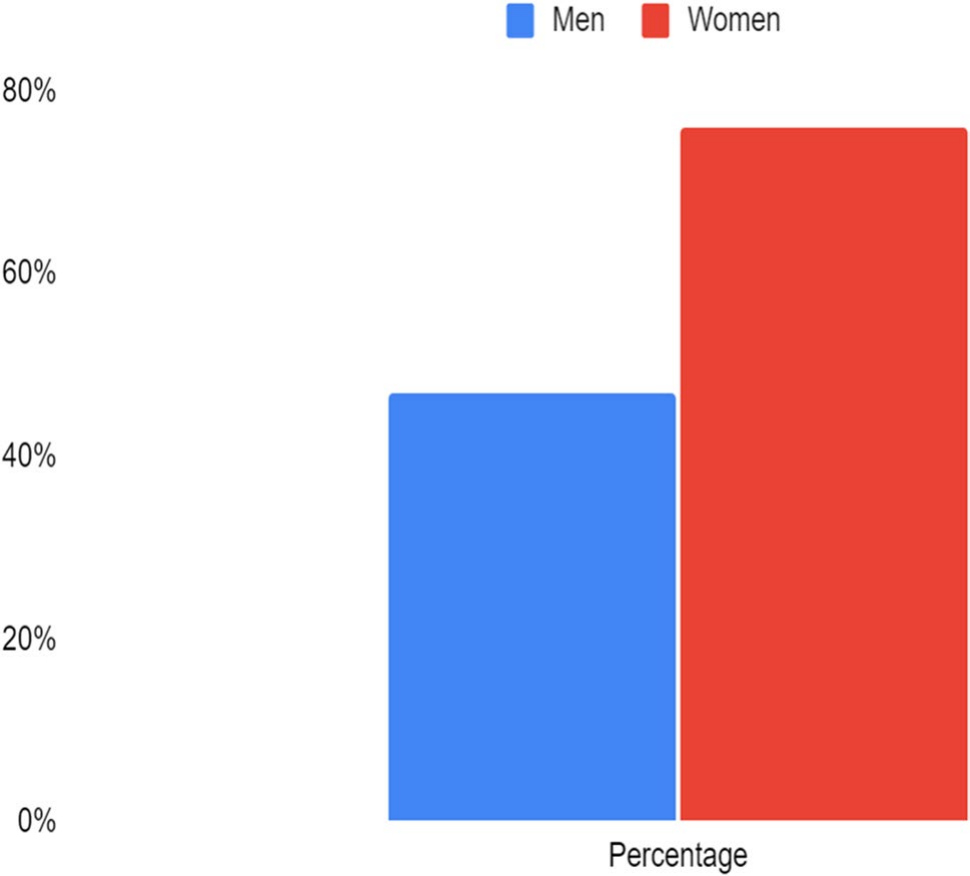
Rate of patients using CPAP during follow up

The ODI was higher in men (*p* = 0.04) (Fig. 1), while neither SpO2 nadir nor the mean desaturation duration differed between the two sexes (*p* = 0.5). In men, both age and BMI were linearly positively correlated with AHI and ODI (r = 0.34, *p* = 0.03 and r = 0.36, *p* = 0.04 for age, r = 0.45, *p* = 0.007 and r = 0.35, *p* = 0.04 for BMI). The mean ESS was below the positivity threshold in both sexes (7 ± 5 points for men, 8 ± 6 points for women) and did not correlate statistically significantly with age, BMI, AHI and ODI (r = 0.1 and *p* = 0.5 for age, r = -0.3 and *p* = 0.06 for BMI, r = -0.005 and *p* = 1 for AHI, r = -0.02 and *p* = 0.9 for ODI). Women had a higher frequency of hypopneas compared to men (*p* = 0.003). CPAP therapy had been prescribed to 25 women and 34 men, but at the follow-up visit there were more women on CPAP therapy than men (76 vs 47%; χ = 5, *p* = 0.02) (Fig. 2). In particular, eighteen men (53%) and six women (24%) had decided to discontinue CPAP therapy on their own due to poor compliance during the night, because of reported air leaks from the mask or because they felt they were unable to sleep for the high pressures. The mean therapeutic pressure used in CPAP did not differ significantly between men and women and did not correlate with AHI (r = 0.2, *p* = 0.4).

## Conclusions

In women, the lower mean age at diagnosis demonstrate that the onset of OSAS occurs earlier in the female sex, given the high post-menopausal risk [1]. In fact, with the onset of menopause, previous literature reported that the prevalence of OSA doubles [12, 13] and that there is a reduction in respiratory drive and an increase in arousals and soft tissue collapsibility compared to premenopausal age [14, 15]. The hypothesis highlighted in previous scientific studies asserts that estrogen and progesterone play a role in the control of ventilation and airway collapsibility during sleep [16]. 17β-estradiol also protects women from the risk of developing OSAS through its antioxidant effects and through stimulation of upper respiratory tract musculature. Therefore, there is an increased susceptibility to OSAS with the onset of menopause and reduced serum levels of these hormones [17]. This finding may suggest the introduction of screening for OSAS through dedicated questionnaires to women around menopausal period, to achieve early diagnosis and avoid long-term complications. The higher frequency of hypopneas compared to men is in agreement with data from previous scientific studies [18] and could be related to the pathophysiological mechanism of low arousal threshold [14, 19]. In fact, postmenopausal women, as reported in some previous scientific studies, have some typical polysomnographic predictors of low threshold arousal [20]: AHI < 30 events per hour, nadir SpO2 > 82.5% and hypopnea frequency > 58.3%. These results were also seen in women in our study. In men, the supine AHI was significantly higher, in agreement with the literature [21]. It is possible to assume that the supine position in men stimulates the tonic activity of the dilator muscles of the upper airways, while it reduces the phasic activity [22]. Previous data in literature show that men develop an elliptically shaped airway in the supine position with the long axis oriented laterally [23]. This anatomical change may generate altered pressure gradients in the velopharynx and therefore increase the propensity to collapse [24]. This finding may suggest the use in men of positional devices that avoid supine position, especially when CPAP compliance is low. Furthermore, in men, the AHI and ODI were higher with increasing BMI and age, showing how obesity and senescence have a role in the pathophysiological mechanisms especially in the male sex. This is confirmed, for example, by AHI reduction after weight loss, both through bariatric surgery and/or intensive lifestyle intervention [25, 26]. This finding may suggest the introduction of mass screening tests for OSAS in men with obesity, as well as the use of dietary interventions as first-line treatment in conjunction with CPAP therapy to reduce the severity of apneas. The low therapeutic CPAP adherence in men is a finding that can be explained at least in part by the higher average age and thus to an underestimation of long-term complications. In addition, also due to facial anatomical factors, men may have a more difficult adaptation to facial and/or nasal masks. This result, consequently, implies more attention to be paid to male patients both at the time of CPAP adaptation and during their follow-up. In addition, the implementation of telemedicine to follow-up these patients more intensively over time may be a right move to increase therapeutic adherence to CPAP [27], as well as also the correct disclosure of OSAS severity and its complications, also working on the emotional-behavioral sphere [28]. The ESS did not show significant specificity for the two sexes and its mean value was below the threshold of positivity in both sexes, underlining the limitations of these questionnaires regarding excessive daytime sleepiness (EDS). ESS sensitivity and specificity may increase with the support of patient's partner in the completion of the questionnaire and with physician supervision to avoid misunderstandings in the interpretation of the test. Moreover, the ESS is a test that assesses only a part of the patient's symptomatology; in fact, the literature reports that EDS is present in only 40% of patients with OSAS [29]. In addition, literature reported that ESS is not strongly associated with EDS in the female sex; in fact, women often have an ESS score < 10, probably due to a different threshold for perceived sleepiness [30].

The strength of the present study is that it has comprehensively investigated all the factors that may determine the gender differences found in clinical practice with OSAS patients. In fact, having collected data not only on polygraphic patterns but also on epidemiology and symptomatology (EDS), allowed us to identify several risk factors for OSAS that are more present in one sex than the other. In addition, we also investigated the follow-up behaviour of men and women, measuring therapeutic adherence to CPAP, so that we could also make a gender comparison on the consideration that these patients have for the pathology.

Possible limits of the present study lie in its retrospective and observational nature. As a pilot study, its small sample size could affect the generalisability of the results and the application of the findings in daily clinical practice. Furthermore, this study lacks measurements of anatomical factors (neck circumference, diameter of the oropharynx and neck fat on MRI), hormonal factors (serum dosage of female sex hormones such as luteinising hormone or follicle-stimulating hormone and male sex hormones such as testosterone or dehydroepiandrosterone) and oesophageal pressure to quantify the arousal threshold.

In conclusion, this study showed that, especially in women, BMI cannot be uniformly used as the main risk factor for OSAS severity. Moreover, in women, it is essential to suspect the syndrome in the period following the onset of menopause. The typical polysomnographic pattern in women has been that of a high hypopnea/apnea (HI/AI) ratio, while in men there is a high supine AHI and high ODI. In men, it is important to assess AHI in the supine position, given the possibility of using positional therapy along with or as an alternative to CPAP, considering the lower therapeutic adherence compared to women.

## Data Availability

The dataset used for our analysis are available upon demand to the corresponding author of this study.
